# Viral Hijack of Filamentous Surface Structures in Archaea and Bacteria

**DOI:** 10.3390/v13020164

**Published:** 2021-01-22

**Authors:** Colin Tittes, Sabine Schwarzer, Tessa E. F. Quax

**Affiliations:** Archaeal Virus-Host Interactions, Faculty of Biology, University of Freiburg, Schänzlestrasse 1, 79104 Freiburg, Germany; colin.tittes@biologie.uni-freiburg.de (C.T.); sabine.schwarzer@biologie.uni-freiburg.de (S.S.)

**Keywords:** archaeal virus, phage, viral entry, pilus, flagellum, archaellum

## Abstract

The bacterial and archaeal cell surface is decorated with filamentous surface structures that are used for different functions, such as motility, DNA exchange and biofilm formation. Viruses hijack these structures and use them to ride to the cell surface for successful entry. In this review, we describe currently known mechanisms for viral attachment, translocation, and entry via filamentous surface structures. We describe the different mechanisms used to exploit various surface structures bacterial and archaeal viruses. This overview highlights the importance of filamentous structures at the cell surface for entry of prokaryotic viruses.

## 1. Introduction

Microbial viruses are extremely abundant and diverse. They are estimated to outnumber their hosts with a factor 10 in some environments and are found in a huge range of habitats [1]. Viruses can infect members of the three domains of life: archaea, bacteria and eukaryotes. Each domain of life is infected by a distinct group of viruses [2]. Archaeal viruses display particularly diverse virion (viral particle) morphologies [2,3]. Many archaeal viruses, especially those infecting crenarchaea, show morphologies that are not found for those infecting bacteria or eukaryotes. Virion morphologies have implications for their ultimate function: delivery of the viral genome in the host cell (entry) [4,5]. For example, head-tail virions are able to eject their genome, while their capsid remains largely intact, whereas rod-shaped virions have to disassemble completely to free their genome [6,7]. Spindle shaped viruses likely also allow for DNA ejection, which is affected by their capability of transitioning from lemon-shaped capsids to tubular structures, where the DNA is under higher pressure [8,9,10,11]. In contrast to non-enveloped viruses, virions covered in a lipid membrane additionally need to fuse their membrane with cellular membranes before the genome is released. Despite the large genomic and morphological variety between viruses, their viral entry strategies underlie the same basic principles [4]. In all cases, the major barrier virions need to cross is the cell envelope to deliver their genome into the cytoplasm, where it has access to host resources and replication machinery. Often this is a two-step process, in which the first cellular contact of a virion is reversible, followed by specific irreversible adsorption to a secondary surface receptor [4,12]. Reversible and irreversible adsorption can occur at the same receptor, but more generally involve two different receptors [4,12].

**Figure 1:**
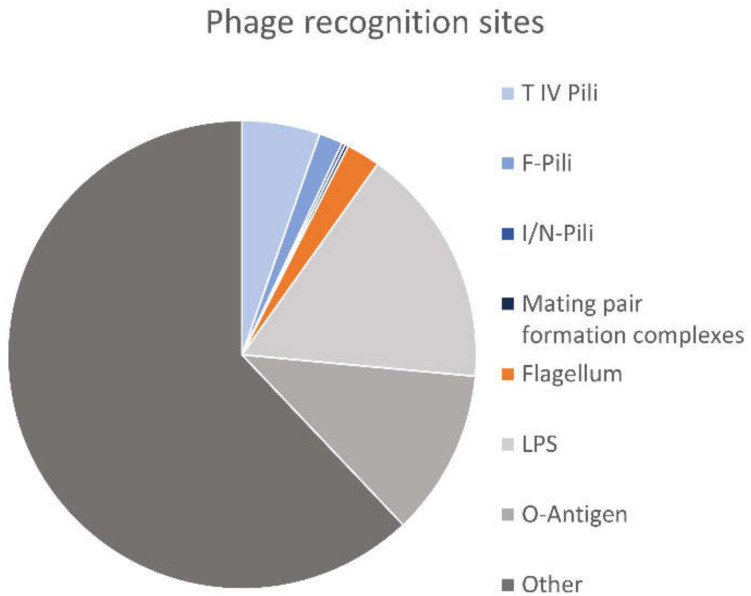
Comparison of recognition sites of bacterial viruses. Pie chart based on the database introduced [13]. Filamentous surface structures are a comparatively rare recognition site (~7.5% of total). As the database does not distinguish between primary and secondary recognition sites, some phages that use different primary and secondary receptors are included twice (once for each receptor).

First, virions must approach the cell envelope of their future host cell and upon reaching the cell surface they transfer their genome or the entire virion across the cell envelope. As viruses are inert and have no means to move themselves in the direction of the cell surface, it is advantageous for them to ride on filamentous surface structures in the direction of the cell surface. The cell envelopes of bacteria and archaea are covered with several different filamentous surface structures, which present potential initial viral attachment sites. Numerous viruses have developed strategies to utilize these surface filaments in an initial step of the entry process (for an overview, see: [12,13]). Such strategies are used by a minority of all viruses. Only about 7.5% of bacterial virus recognition sites are filamentous surface structures (Figure 1) [13]. By comparison, lipopolysaccharide (LPS) is one of the most frequently used viral binding sites, making up ca. 16.6% of all bacterial virus recognition sites (Figure 1). Ca. 50.1% of all binding sites involve sugar moieties [13]. Use of filamentous surface structures is much rarer among viruses infecting Gram-positive bacteria than among those infecting Gram-negatives, with one recorded instance of such a virus (*Bacillus* virus PBS1) using the flagellum as a recognition site [14]. Instead, these viruses most frequently recognize sugar moieties of cell wall teichoic acids [13].

Most families of bacterial viruses contain members that use filamentous surface structures as initial binding sites. However, some families, such as *Inoviridae* and *Leviviridae* almost exclusively harbor members that rely on surface filaments for attachment. Thus, the use of surface filaments for entry seems to be linked with, but not exclusive to, certain viral families. This phenomenon is not consistent for all members of a family, and in most cases, the filament binding viruses represent only a small fraction of all family members.

The aim of this review is to provide a general impression of the mechanisms that are employed by bacterial and archaeal viruses to approach their host cell via filamentous surface structures. We focus on bacterial viruses (also called phages) for which several such mechanisms have been explored in detail [15,16], and also on archaeal viruses, which were only recently shown to interact with filamentous cell structures. Importantly, we do not intend to list all viruses that are reported to attach to surface filaments. Such information is readily accessible via online databases [12,13]. Instead, we discuss interesting cases from bacterial and archaeal viruses, of which the underlying molecular mechanisms have been studied.

## 2. Attachment to Surface Filaments by Bacterial Viruses

**Figure 2:**
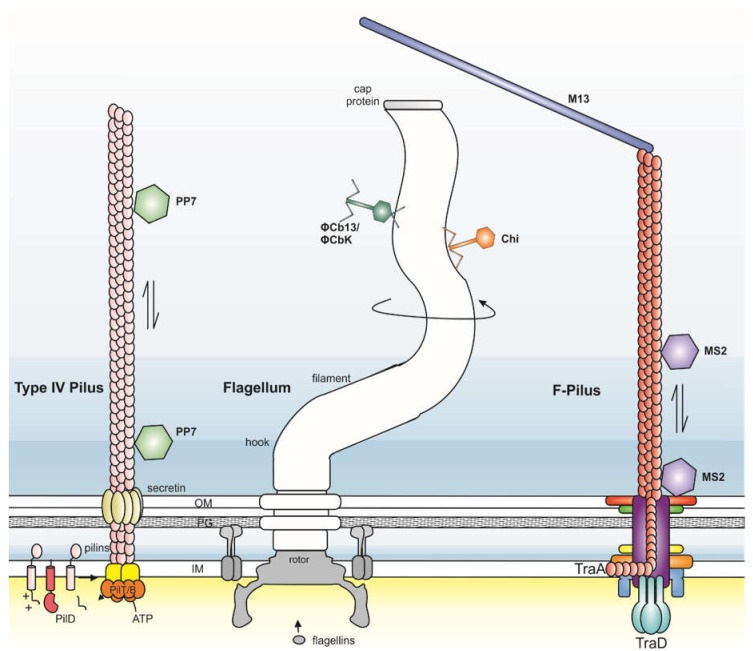
Schematic representation of viral interaction with filamentous structures on the surface of bacteria. The scheme represents the surface of a Gram-negative bacterium. From left to right a type IV pilus (Type II secretion), flagellum (Type III secretion) and F-pilus (Type IV secretion) are shown. (Left) Type IV pili consist of pilins (pink), which are N-terminally processed by PilD and inserted at the base of the growing filament with the help of the ATPase PilB. Secretins allow the filament to cross the outer cell membrane. The pilus can undergo circles of extension and retraction with help of the retraction ATPase PilT, which is necessary for twitching motility. Several viruses, such as PP7 are able to bind to the Type IV pili and when the pilus retracts, are pulled towards the cell surface, where they can interact with a secondary receptor on the cell surface (not shown). This structure is not hollow. (Mid) The flagellum is a rotating filament that is responsible for swimming motility in liquid medium. New subunits, flagellins, travel through the hollow filament and are added at the tip. Rotation relies on a proton motive force. Several viruses can attach to the flagellum, such as Chi (orange) with its tail fibers. ΦCb13 and ΦCbK (green) use their head-filaments to attach to the flagellum. In both cases, the active rotation of the filament supports the translocation of the virions along the length of the filament in the direction of the cell surface according to the nut and bolt model. (Right) The F-pilus is encoded by the F sex plasmid and is involved in conjugation. The hollow pilus dynamically extends and retracts to find a potential recipient cell and draw it into direct contact with the donor cell. Several viruses bind to the F-pilus, including members of the *Inoviridae*, such as M13 and the *Leviviridae*, such as MS2. M13 binds the tip of the F-pilus, while MS2 binds along the length of the filament. Retraction of the F-pilus pulls MS2 in the F-pilus channel, which leads to the disruption of the virion and disconnection of the F-pilus. The ATPase TraD couples the F-plasmid substrate to the Type IV Secretion machinery. It is required for MS2 gRNA delivery but not for delivery of Qβ gRNA [33,34]. OM, outer membrane. PG, peptidoglycan. IM, inner membrane.

The bacterial cell envelope generally consists of one (Gram-positive) or two (Gram-negative) cell membranes and a cell wall of murein, also called peptidoglycan. Additionally, bacteria can present a variety of filamentous structures at their surface such as the bacterial motility structure (the flagellum), F-pili, type I-pili, and type IV pili (T4P) that are involved in twitching motility (Figure 2). All of them are produced by different secretion pathways, have different structures, and are made of different protein components. Some of these structures, such as F-pili and T4P, have the ability to extend and retract, helping attached viruses to move towards the cell surface [17,18].

### 2.1. Flagellar Viruses

Flagella are the bacterial motility structures, which are responsible for swimming movement in liquid. The structure consists of over 30 different proteins that build a motor structure and a long filament made of thousands of copies of the main protein component (flagellins) [19]. The filament is hollow and flagellum subunits travel through the filament during assembly. Growth then occurs at the tip, which is covered by a cap structure [19]. The chemotaxis system works in concert with the flagellum in order to achieve directed movement and this system can determine if the flagellum rotates clockwise (CW) or counterclockwise (CCW) [20].

A number of Caudoviruses attach to bacterial flagella (Figure 2). The most well-studied is phage χ infecting *Eschericha coli* and *Salmonella* sp. [21,22], while other examples are ΦCB13 and ΦCbK of *Caulobacter crescentus* and phage 7-7-1 of *Agrobacterium* sp. strain H13-3 [23,24]. There is furthermore evidence that *Bacillus* virus PBS1 uses the flagellum of *Bacillus subtilis* as its recognition site [14], indicating that this strategy is not restricted to Gram-negatives. Motility of the host was identified early on as a key requirement for infection of phage χ. The tail fiber of χ binds flagella randomly along the length of the filament [21,25]. Binding to the flagellum is reversible [21]. Binding also takes place when flagella are sheared off the cell [21,25]. However, to translocate to the cell surface, active rotation of flagella is required, although the cell body itself does not need to move. This was shown by infection of cells with straight flagella, which still rotate but do not result in cell movement [21,25,26]. These findings can be explained by the nut-on-a-bolt mechanism for virus transportation along the filament posited by Berg and Anderson [27]. This hypothesis assumes that the tail fiber of χ fits the grooves formed by helical rows of flagellins and that active rotation of the flagellum forces the virus to follow the grooves similar to a nut following the threads of a bolt [27,28]. Corresponding with this hypothesis is the finding that χ only efficiently infects in case of flagella with grooves of a certain size [28]. At high multiplicity of infection (MOI), multiple empty virion particles could be found at the base of rotating flagella. This suggests that when the particles reach the base of the flagellum, they bind irreversibly and deliver their genomic DNA in the host cell [21].

Next, a survey of χ adsorption was performed in deletion strains of chemotaxis mutants, which cause cells to alter their swimming behavior (i.e., more smooth or tumbly) [22]. This analysis showed that pausing of flagellar rotation inhibited χ adsorption and that adsorption was highest in strains that rotated the flagellum incessantly [22]. Phage χ can most efficiently infect in case of CCW rotation of the flagellum filament, resulting in smooth swimming (Figure 2) [28]. A similar impact of rotation direction was found for infection of the viruses ΦCB13 and ΦCbK of *C. crescentus* [23]. For these viruses, the efficiency of infection was also higher on cells that had a CCW bias, in line with the nut-on-a-bolt model [23]. Importantly, ΦCB13 and ΦCbK do not attach to the flagella with their tail fibers like χ, but instead use a flexible and variable-length head-filament to slide in the groove of the flagellum (Figure 2) [23]. It has been hypothesized that CW rotation would cause virions to move away from the cell surface. Additionally, during CW flagellar rotation *C. crescentus* moves forward in liquid, causing phage particles to be dragged by the cell [23]. In contrast, CCW rotation results in backwards motion, which helps the virions orient their tail fibers towards the base of the flagellum, favoring attachment with their tail fibers to their secondary receptor located at the cell surface [23].

After reversible attachment to flagella, bacterial viruses bind different secondary receptors at the cell surface. For example, phage 7-7-1 of *Agrobacterium* sp. binds lipopolysaccharide (LPS) components, namely the O-specific polysaccharide-containing galactose [29]. The authors assume that after binding to this secondary receptor, the virus cleaves specific sugars on the polysaccharide chain, shortening the chain and gaining access to the cell membrane whereupon it punctures the outer membrane, degrades the peptidoglycan layer and ejects its DNA into the host cell [29]. Such LPS degrading enzymes are usually part of the virion, as in the case of *Salmonella* infecting phage P22. Interestingly, the flagellum binding *Caulobacter* phages ΦCB13 and ΦCbK need TAD pili (T4P) for successful infection and are suggested to transfer their genome through pili portals (i.e., the membrane spanning parts of the pilus assembly and scaffolding) [23]. Their genome entry might occur in a similar fashion as that of T4P specific phages, which has unfortunately not yet been described. Thus, these viruses represent a rare case in which the entry occurs via pili portals, although the viruses did not use pili as primary receptors.

Phages ΦCB13 and ΦCbK are siphophages and have a flexible tail, which may facilitate binding of the tail fibers to the surface receptor, the TAD pili, which are found at the cell pole in the vicinity of the flagellum in *C. crescentus* [30]. As other phages also contain head-filaments, it might be possible that the role of head-filaments in attachment to flagella is widespread [23,31]. The head-filament might represent a distinct advantage as it physically separates the binding sites for the first and secondary receptor. In case of virions that require the tail fibers to bind both the flagellum and secondary receptor, successful contact with the secondary receptor is only possible if either the phage tail fiber temporary detaches from the flagella, or if auxiliary tail fibers make the contact. This would also limit the radius from the flagellum in which the secondary receptor can be present [32].

In short, there are several different bacterial viruses that use the flagellum as primary attachment point, and the rotation of the filament is required for translocation of the virions to the cell surface.

### 2.2. Bacterial Viruses Attaching to Type IV Pili

T4P are adhesive filaments that are produced by a wide range of Gram negative bacteria and the subunits (pilins) of which are secreted via a similar mechanism as the type II secretion system (T2SS). They can mediate attachment to surfaces, biofilm formation, twitching motility, and DNA exchange [17,35]. T4P are widespread in bacteria and are defined based on their assembly mechanism [35,36]. They consist of thousands of copies of the major pilins, and in addition a low amount of minor pilins, which are likely positioned at the top of the assembled pili [37]. T4P pilins are made as preproteins, N-terminally processed and transferred across the cytoplasmic membrane by the Sec-pathway [38,39]. Pilins are then extracted and assembled into the pilus filament by an ATPase [36]. A special characteristic of the T4P is that the majority of these filaments is capable of retraction through the cell wall [17,40]. This property is essential for so-called twitching motility, where cells glide over a surface [41].

Viruses make use of this capability of retraction, as this enables them to be pulled towards the cells surface when they are attached to pili (Figure 2) [42]. Already in the 1970s, electron microscopy of infected cells enabled the visualization of shortening of *Pseudomonas* T4P after virus attachment [43]. The viruses were found to bind along the length of the *Pseudomonas* pili and were pulled towards the cell surface when the pili retracted, which suggest that viruses bind the major pilin [44]. Many *Pseudomonas* viruses use two main receptors as binding sites: the lipopolysaccharides (LPS) and T4P [42,45]. Glycosylation of the major pilin of T4P of *Pseudomonas* was found to be an important factor in pilus recognition [45]. As the exact role of pilus glycosylation is not clear, the authors propose that differential glycosylation represents an efficient strategy to avoid viral infection [45]. 

Thus, T4P are the primary receptor for a small number of diverse phages. Retraction of the pili enables virions to reach the cell surface where they can bind irreversible to their secondary receptor.

### 2.3. Viruses Attaching to Bacterial F-Pili

Bacterial conjugation systems are members of the type IV secretion system (T4SS) superfamily (not to be confused with T4P) [46]. In Gram-negative bacteria, conjugation machines comprise not only a mating channel but also a conjugative pilus used to attach to other cells [47]. The best-known example of such a conjugation machine is the F-pilus, encoded by the F sex plasmid. This is a hollow, filamentous, and dynamic appendage [47]. In the “mate-seeking” mode, the pilus dynamically extends and retracts to find a potential recipient cell and draw this into direct contact with the donor cell [18,48].

Many viruses can bind these F-pili. Icosahedral RNA viruses such as f2/MS2/R17 and Qβ (belonging to the *Leviviridae*) bind F-pili specifically along the length of the pilus [49], whereas several ssDNA filamentous bacterial viruses from the family *Inoviridae* bind the F-pilus at the tip (Figure 2) [50]. The secondary receptor of *Inoviridae* is the TolQRA complex of inner membrane proteins, which is highly conserved in Gram-negative bacteria. TolQRA belongs to the larger trans-envelope Tol-Pal complex, which is involved in maintenance of cell envelope integrity [51,52]. The minor coat protein (pIII) located at one end of the filamentous phage particle is responsible for pilus tip interaction [53]. Its two N-terminal domains bind both to the F-pilus tip and to the secondary receptor, while the C-terminal domain is involved in virion uncoating and DNA entry into the host cell [53,54]. pIII is the most diverse virion protein amongst *Inoviridae* viruses, and often has no significant homology between distantly related viruses. Usually, the protein is only recognized by its size and position in the viral genome. The function of pIII was studied in detail for Ff phage, and it was shown to undergo a conformational change upon F-pilus binding, leading to exposure of the TolA binding site of pIII [55,56,57]. Similar results were found for the filamentous phage fd, which also attaches to F-pili [55].

In absence of the F-pilus or the F-pilus binding domain on pIII, the infectivity of viruses decreases by several orders of magnitude, but is not completely abolished [58]. However, the secondary receptor TolQRA and its cognate binding site are absolutely essential for infection [56,59]. As the F-pilus undergoes spontaneous oscillatory extension and retraction cycles, filamentous virions that have attached to the F-pilus tip will be drawn to the cell surface. By an unknown mechanism, they cross the outer membrane and arrive at the periplasmic space where they can interact with the periplasmic domain of TolA. Eventually, this results in entry of the phage ssDNA into the cytoplasm and integration of the major coat protein into the inner membrane [54,60,61].

The pIII of phage IF1 functions slightly different as that of Ff phage, as the binding site for the secondary receptor is permanently accessible [62]. This virus binds to IncI plasmid encoded pili instead of F-pili using its version of the pIII, to bind both IncI-pili and TolA-C [62]. IncI-pili are T4 pili and thus unrelated to F-pili [63]. IF1 binds to IncI-pili and, like phage Ff, may use pilus retraction to approach the host surface. 

More insight into the key mechanism by which the F-pilus promotes viral entry into the host cell was recently provided by the study of the ssRNA *Levivirus* MS2, which infects F+ *E.coli* cells [34]. This virus encodes four proteins: Mat, involved in host recognition, the coat protein, an RNA dependent RNA replicase and the lysis protein. The tailless virions consists of 178 copies of the coat protein, and only one of Mat [64]. The Mat protein allows virions to adsorb to the side of the F-pilus. It was shown that retraction of F-pili and associated virions could trigger the detachment of F-pili. The F-plasmid encoded coupling protein TraD is an ATPase situated at the channel entrance that couples the F-plasmid substrate to the T4SS for conjugative transfer [65]. TraD is not required for F-pilus formation [65]. Interestingly, MS2 binding to F-pili from Δ*traD* F+ strains still resulted in detachment of F-pili [34]. However, the gRNA (genomic RNA) of the virus did not enter the cytoplasm, suggesting that the F-pilus detachment occurs at an early step of MS2 interaction with the F-pilus. Qβ on the other hand was still capable of infecting Δ*traD* mutants [33]. A model has been proposed in which MS2 binds to the F-pilus and via pilus retraction is able to interact with the distal end of the T4SS channel (Figure 2) [34]. Continuous pilus retraction forces the Mat protein–gRNA complex from the virion into the T4SS channel [34]. A previous cryo-EM study has also suggested that the dynamics of the F-pilus is the driving force behind MS2 genome delivery, as it causes attached viruses to get stuck and then pulls the viral genome out of the virions via the Mat protein [66]. This retraction force that is blocked by the MS2 particle, likely enforces a torsional stress leading to the detachment of the F-pilus. In the same study, other F-pilus binding viruses were analyzed as well, showing that the ssRNA virus Qβ also induces the detachment of F-pili, although to a lower extent [34]. In contrast, the Inovirus M13, which binds to the tip of the F-pilus, did not cause F-pilus detachment, suggesting that this entry mechanism might be conserved amongst ssRNA viruses, but not for Inoviruses [34].

In summary, F-pilus binding viruses are being transported to the cell surface via F-pilus retraction. The entry mechanism of Inoviruses is unknown, while that of Leviviruses relies on the physical force of F-pilus retraction that pulls the gRNA into the T4SS channel.

## 3. The Use of Filamentous Surface Structures by Archaeal Viruses

Entry mechanisms of archaeal viruses are relatively unexplored, and they might be quite different from those of bacterial viruses, as the cell surface environment differs between these two domains of life. Archaea do not have a cell wall of murein. Instead, many archaea are covered in a protective Surface (S) layer, built of multiple copies of 1–2 glycosylated proteins that form a semi-crystalline sheet [67,68,69]. In several archaea, the S-layer is the sole constituent of the cell wall, or the S-layer forms the cell wall in combination with other polymers. The archaeal cell envelope, like that of bacteria, is covered with surface filaments. The majority of the archaeal surface filaments have homology to bacterial T4P [70]. The filament forming proteins are N-terminally cleaved by a signal peptidase, before the subunits are added to the growing filament structure, in a similar fashion as bacterial T4P. Archaeal T4P are involved in attachment, biofilm formation, DNA exchange and motility [71]. Archaea employ adhesive pili for attachment and biofilm formation [72,73,74]. These pili likely do not retract, as twitching motility has not been observed and homologs to the bacterial retraction ATPase have not been found in archaea [75].

Archaea use a rotating T4P, the archaellum, for swimming motility. It has a similar function as the bacterial flagellum, but no structural similarity [76]. Pili and archaella are heavily glycosylated [67,73,77]. In addition, several archaea possess non-T4P, such as the “threads” of unknown protein composition that are seen at the cell surface of thermoacidophilic crenarchaeon *Sulfolobus acidianus* [74].

### 3.1. Entry of Archaeal Viruses

The available information on entry mechanisms of archaeal viruses originates from a handful of model viruses, which primarily infect hyperthermophilic archaea from the genus *Sulfolobales*. As the entry mechanisms of archaeal viruses are not well characterized, there are currently only a few viral receptors identified. A first example is that of the Caudovirus ϕCh1 that infects a halophilic euryarchaeon, and uses its tail fibers to bind to galactose moieties on the surface of its host [78,79]. Another is OppA(Ss), which is an N-linked glycoprotein that specifically binds oligopeptides and was shown to interact with Acidianus two-tailed virus (ATV) minor protein p529 [80]. It is likely that archaeal viruses, like their bacterial counterparts, recognize and bind cell wall components, such as S-layer proteins and sugar moieties.

After adsorption, archaeal viruses with a head-tail morphology likely eject their genomic content in the cell, in a similar fashion as bacterial viruses of this morphology. However, the majority of archaeal viruses has different shapes, and many of them are enveloped [2]. It seems probable that these enveloped viruses need to fuse with the cell membrane to internalize their genome. Indeed, in case of *Pleolipiviridae*, which produce pleomorphic membrane particles, the 57 kDa virion spike protein mediates membrane fusion [81,82]. A fusion event during entry was also suggested for the entry of Sulfolobus Monocaudavirus 1 (SMV), which during entry aligns along the cell surface, followed by flattening of the viral surface [9]. SMV1 was suggested to belong to a superfamily together with other large spindle shaped viruses, such as ATV and Acidianus tailed spindle-shaped virus (ATSV) [83]. Interestingly, the ATSV virion, like some other members of this family (i.e., SMV1) develops long tails extracellularly [8,9,84]. Structural analysis of ATSV led to a model that suggests that such viruses are released in a metastable state, which provides energy for capsid rearrangements that result in the development of tails outside the cell, concomitantly with a reduction of the capsid volume [8]. The reduction of capsid volume increases pressure on the genomic DNA [8]. Host binding by virus tail fibers has been suggested to trigger opening of the particle and DNA ejection due to the internally built pressure [8]. Whether the DNA ejection mechanism of ATSV and the “flattening” of SMV1 during entry are part of the same entry mechanism or both related viruses use different strategies is not yet clear. His1, a virus belonging to the *Fuselloviridae* family that infects *Haloarcula hispanica,* was shown to eject its genome into the host cell via a process that is modulated by external osmotic pressure. Further, it was suggested that cellular processes are required to complete the DNA transfer of His1 [11].

### 3.2. Interaction of Ligamenvirales with Archaeal T4P

**Figure 3:**
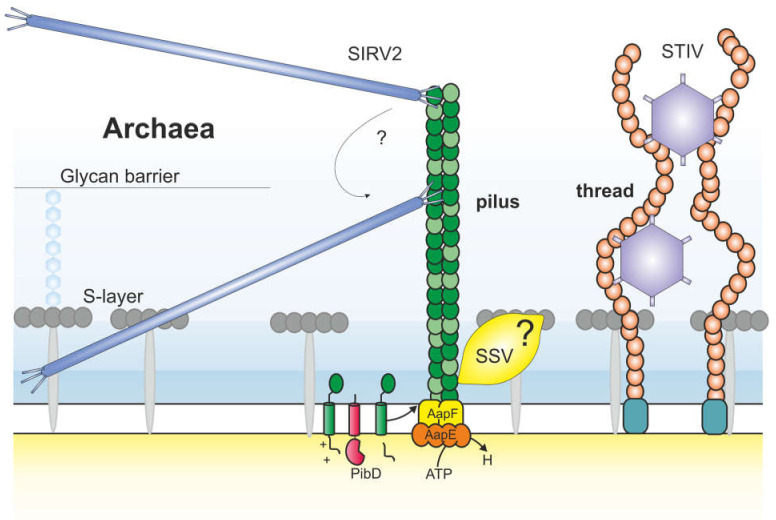
Schematic representation of viral approach of the archaeal cell surface. This schematic is based on the crenarchaeon *Sulfolobus*. *Sulfolobus* has a single membrane and a cell wall of S-layer (gray, surrounded by a glycan barrier (light blue, black line indicates height). It displays different filamentous structures at its surface, which all do not have a hollow interior. In green adhesive T4P are shown, which have homology to bacterial T4P. The pilins are N-terminally processed by PibD before they enter the pilus at the cell proximal end. AapF and AapE have homology to the bacterial T4P proteins PilC (inner membrane platform protein) and PilB (the cytosolic assembly ATPase of the motor complex), respectively. SIRV2 (blue) has a rod-shaped virion of ~900 nm in length and it can bind both at the tip and along the length of the pilus with help of its three tail fibers. It is unknown how the virus translocates along the filament to the cell surface. Retraction of archaeal T4P has not been observed. Adhesive pili are also important for infection of the spindle shaped virus SSV (yellow). Sulfolobus also displays thin non-T4P-like filaments at its surface: threads (orange). The icosahedral virus STIV1 (purple) uses its turrets to bind to these threads. One particle can bind multiple threads and virions get entangled in the filaments. It has been hypothesized that a random walk would lead them to the cell surface.

Recently, it has become clear that viruses also recognize filamentous surface structures of archaea. Whole cell cryo-electron tomography (cryo-ET) has played an important role in this discovery. Already several years ago, it was noted that purified virion particles were in some cases still attached to filamentous cell surface structures, suggesting that virions bound specifically to the filaments. For example, the Acidianus Filamentous Virus 1 (AFV1) infecting the hyperthermophilic crenarchaeon *Acidianus hospitales* contains claw-like structures at the distal end of the virions that were found to specifically bind filamentous surface structures [85]. Both ends of the virion seemed to have equal binding capacity. Similar observations were made for the Rudivirus Sulfolobus islandicus Rod-shaped virus 2 (SIRV2) [86], which belongs to the same order as the Lipotrixvirus AFV1: *Ligamenvirales* [87]. The rod-shaped SIRV2 virion has three tail fibers at each distal end, with which it can interact with filaments presented on the cell surface of its host *Saccharolobus islandicus* (previously *Sulfolobus islandicus*) (Figure 3) [88]. SIRV2 tail fibers at each side of the virion can interact with surface filaments, as was shown with electron micrographs that occasionally revealed one virion attached to two filaments [88]. Whole cell cryo-electron tomography showed initial attachment of SIRV2 to filamentous structures on the surface of *S. islandicus*. To test the specificity of the binding, virions were mixed with isolated filaments from uninfected cells [88]. This showed that the virions bound specifically at the tips of the isolated filament, similarly as seen for filamentous bacterial viruses like M13 [50]. However, when the virion-filament interaction was studied in filaments attached to living cells, only 30% of the virions attached to the tip, while the rest were found along the length of the filament and at the cell surface [88]. This suggests that initial attachment occurs at the tip and filaments need to be attached to living cells in order for the virions to translocate towards the cell surface. Clues as to the identity of the filament to which SIRV2 attaches came later, when genes with homology to T4P encoded by the related archaea *Saccharolobus solfataricus* (previously *Sulfolobus solfataricus*) were found to be important for SIRV2 infection [89]. Specifically, loss of AapF and AapE in *S. solfataricus* resulted in viral resistance against SIRV2 [89]. AapF and AapE are part of the adhesive pilus of *Sulfolobus* and have homology to the bacterial PilB and PilC that form the motor of bacterial T4P [74]. Interestingly, there is an insertion element in the adhesive pilus operon of *S. solfataricus,* which was hitherto predicted not to have an adhesive pilus [74]. Additional evidence for the role of T4P in entry of a different SIRV came by studies of viruses infecting *S. islandicus*. Deletion of the two pilins *pilA1* and *pilA2* of the adhesive pilus of *S. islandicus* resulted in cells without pili that were resistant to SIRV infection. Indeed, it was shown that SIRV virions adsorb to adhesive T4P (Figure 3) [90].

### 3.3. Viral Entry Via Interaction with Archaeal Adhesive Pili

There is strong support for the notion that SIRV binds archaeal adhesive pili, although it cannot be ruled out that SIRV primarily adsorbs to other filamentous surfaces structures and requires the adhesive pili only for secondary adsorption and entry. The mechanism by which pili could contribute to efficient viral entry is not directly evident. Archaeal T4P likely do not retract, as this was never observed and a retraction ATPase is not present in any sequenced archaeal genome [70]. Thus, archaeal viruses likely rely on translocation mechanisms which allow pili binding viruses to move in the direction of the surface along the length of the filament. Cryo-EM indicates that the SIRV2 tail fiber protein (ORF1070) is the most plausible candidate to interact both with the tip and with the length of the filament [88,91,92]. This could be a two-step process in which tip-binding induces a slight conformational change in the pilins in the filament, which could enhance the affinity of SIRV2 for the length of the pilus in living cells. As rudiviruses have stiff rod-shaped virions, tip-bound virions cannot flexibly wrap around the pilus to bind the same filament again at a location closer to the cell surface with the tail fibers at its distal end. Therefore, a possible translocation mechanism might involve recurrent rounds of binding and release of the same tail fibers.

### 3.4. Entry of Spindle Shaped Viruses

The infectivity of several Sulfolobus Spindle shaped viruses (SSV) belonging to the *Fuselloviridae* was reduced on pilin deleted strains, similar as for SIRV (Figure 3) [90]. However, SSV9 did not seem directly to adsorb to pilins [90]. In a search for the receptor, the role of the S-layer was tested as it was previously shown that S-layer is important for infection of the related SSV1 [93]. SSV9 could still efficiently infect an S-layer deletion mutant, indicating that SSV9 is dependent on a yet unknown receptor [90]. The role of adhesive pili in entry of SSV has not been resolved.

### 3.5. Binding of Icosahedral Archaeal Viruses to Archaeal Surface Filaments

Cryo-ET revealed that the Sulfolobus turreted icosahedral virus 1 (STIV1) binds filamentous structures on the surface of its host *S. solfataricus* [94]. This icosahedral virus is member of the *Turriviridae*, contains an internal membrane and its major capsid protein has a double jellyroll fold, which places STIV within the adenovirus-PRD1 viral lineage [95,96]. STIV1 presents turret-like assemblies at the five-fold vertices. These are pentameric assemblies composed of three viral proteins, A55, A223, and C381 [96,97,98]. The filament binding occurs with help of these turrets. Whole cell cryo-electron tomography revealed that filament interaction was specifically observed via the side of the turrets, corresponding to the cleft between the second and third domain of turret protein C381 [94]. Interestingly, cryo-EM structures of STIV1 virions showed that the particles from some preparations contained ‘petal’ structures consisting of the C557 protein that covered the turrets [95,98]. These petals are not present on all virions [95,98]. The petals completely mask the binding site of the filaments on the turret protein. Therefore, the petals were hypothesized to be a maturation factor, which ensures that upon release STIV1 particles do not directly bind filaments of the cell that they just lysed [94].

As the STIV virions contain multiple turrets, filament interaction was often observed between one particle and several filaments. The STIV1 virions sometimes appeared completely entangled in the filamentous surface structures [94]. The identity of the filaments is not known, but with an average diameter of ~7 nm and high flexibility, they are likely threads [94]. Threads are not T4P, and their protein composition is still unknown [71]. C381 is diverse between STIV1 and its close relative STIV2 [99]. It has been suggested that the difference in C381 between STIV1 and STIV2, might result in STIV2 binding to other filaments, such as archaella, although this has not been tested experimentally [94].

STIV1 was found to undergo multiple interactions along the length of the filaments. Unfortunately, the current cryo-ET data is not sufficient to reveal a mechanism by which the STIV1 virions could translocate towards the cell surface. As STIV1 particles become entangled in the filaments, it has been speculated that the STIV1 particles behave like burr in a dog’s coat that stochastically moves through the fur until it becomes trapped against the skin [94].

## 4. Conclusions

Bacteria and archaea display a range of different filamentous structures at their cell surface, which serve important functions. Viruses hijack these structures and increase their chances of successful infection by binding or moving along them to the cell surface. Even though it was over 50 years ago that viruses were first shown to attach to filamentous surface structures, the molecular mechanisms underlying translocation of the particles and entry of the viral genome are not completely clear yet. Unfortunately, the scarce and valuable available knowledge for bacterial viruses cannot be extrapolated to archaeal viruses, as the surface filaments are fundamentally different between these two domains of life. It will be of high interest to learn how archaeal adhesive pili support viral translocation in the absence of retraction. Furthermore, the discovery of an archaellum binding virus could help to shed light on the translocation system along the archaellum in comparison to that of the flagellum. Another open question is how evolutionary pressure of viruses shapes the cell surface, and specifically the presentation of filamentous structures. Most filamentous structures offer advantages under certain growth conditions, but are not essential, which offers the possibility to downregulate them in case of viral threat. Environments with high abundance of filament binding viruses might select for cells that do not display filaments at their surface. Similarly, glycosylation of surface filaments is not essential for several organisms, but could provide an advantage because it masks viral binding sites [45]. The ongoing arms race between viruses and cells provides selective pressure that results in the alteration of viral receptors at the cell-surface to avoid viral adsorption. Similarly, it will result in selection of cells that have masked binding sites, for example by glycosylation. Several archaea encode multiple pilins and archaellins as ecoparalogs (i.e., each of these proteins can build the filament individually and is expressed under different conditions) [100,101]. This property allows cells to alter the constituents of their filamentous structures, without downregulating the structure altogether. This likely provides an advantage in the presence of viruses that recognize specific pilins or archaellins.

Conclusively, the study of viral entry does not only address questions about complexity of viral mechanisms, it also helps to gain insight into composition and functioning of the bacterial and archaeal cell surface [43,102,103].
